# Chaihuang weikuining protects against ethanol-induced gastric ulcer through modulation of gut microbiota and inhibition of JAK1/STAT3 signaling

**DOI:** 10.3389/fmicb.2026.1924725

**Published:** 2026-10-27

**Authors:** Ai Fu, Rui Guo, Keyi Wang, Hui Zhuge, Gaoxiang Ying, Wenjun Ma, Qinchao Ding, Songtao Li, Jianfeng Bao, Wangqin Yu

**Affiliations:** 1 Institute of Hepatology and Epidemiology, Affiliated Hangzhou Xixi Hospital, Zhejiang Chinese Medical University, Hangzhou, Zhejiang, China; 2 School of Public Health, Zhejiang Chinese Medical University, Hangzhou, Zhejiang, China; 3 School of Basic Medical Sciences, Zhejiang Chinese Medical University, Hangzhou, China

**Keywords:** chaihuang weikuining, gastric ulcer, gut microbiota, inflammation, JAK1/STAT3

## Abstract

**Background:**

Chaihuang weikuining (CHWKN) is a traditional Chinese medicinal formula with reported anti-ulcer activity. However, the underlying mechanisms remain unclear. This study aimed to investigate the protective effects of CHWKN against ethanol-induced gastric ulcer (GU) and to explore the role of gut microbiota in mediating these effects.

**Methods:**

An ethanol-induced GU model was established in rats. Histological staining (H&E and PAS) and biochemical analyses were used to evaluate gastric injury. Antibiotic (ABX) treatment and fecal microbiota transplantation (FMT) were performed to determine the involvement of gut microbiota. 16S rDNA sequencing was conducted to analyse microbial composition.

**Results:**

CHWKN markedly alleviated ethanol-induced gastric mucosal injury, as evidenced by improved histopathological features and restored of gastric function. CHWKN significantly reduced myeloperoxidase (MPO) and pro-inflammatory cytokines (IL-1β, IL-6, TNF-α, and LPS) while increasing IL-10 levels, accompanied by suppression of *JAK1/STAT3* signaling. Notably, depletion of gut microbiota abolished the protective and anti-inflammatory effects of CHWKN. Conversely, FMT from CHWKN-treated donors (the donor rats treated with ethanol and CHWKN) restored gastric mucosal integrity and reduced inflammation in recipient rats. Furthermore, CHWKN remodeled the composition of gut microbiota by increasing potentially beneficial bacteria and reversing ethanol-induced dysbiosis.

**Conclusion:**

These findings demonstrate that CHWKN exerts protective effects against ethanol-induced GU by modulating of gut microbiota and subsequently inhibiting of *JAK1/STAT3*-mediated inflammation. This highlights a microbiota-dependent mechanism underlying its therapeutic action.

## 1 Introduction

Gastric ulcer (GU) is a common gastrointestinal disorder characterized involving disruption to the gastric mucosal barrier, leading to epithelial damage, bleeding, and, in severe cases, perforation (Arshad et al., 2025; Lanas and Chan, 2017). Despite advances in clinical management, GU remains a significant global health burden. Its pathogenesis is multifactorial, involving Helicobacter pylori infection, non-steroidal anti-inflammatory drug use, and excessive alcohol consumption, contribute to its pathogenesis (Arshad et al., 2025). Ethanol-induced gastric injury is a widely used as a classical experimental model, as it rapidly induces mucosal damage through oxidative stress, epithelial barrier disruption, and inflammatory responses (Rahman et al., 2020). Accumulating evidence suggests that inflammation plays a central role in the development of gastric mucosal injury (Yan et al., 2025; White et al., 2015). Ethanol exposure triggers the release of pro-inflammatory cytokines, such as interleukin (IL)-1β, IL-6, and tumor necrosis factor-α (TNF-α), which leads to the amplification of inflammatory cascades and tissue damage (Wang et al., 2010). The Janus kinase/signal transducer and activator of transcription (JAK/STAT) pathway is a key regulator of inflammatory signaling, and its activation has been associated with gastric mucosal injury and ulcer progression (Agashe et al., 2022; Hu et al., 2021). However, the upstream mechanisms that regulate inflammatory signaling in GU remain incompletely understood.

Recent studies have emphasized the critical role of gut microbiota in gastrointestinal diseases. Beyond its established involvement in intestinal disorders, emerging evidence indicates that gut microbiota also influences gastric mucosal homeostasis and inflammatory responses via the gut-stomach axis (Shabani et al., 2025; Aldriwesh et al., 2026). Specifically, substantial evidence suggests that gastric ulceration is frequently accompanied by gut microbiota dysbiosis, with a marked reduction in both microbial diversity and abundance in patients with gastric ulcers, especially, Firmicutes, Actinobacteria and Lactobacillus (Chen et al., 2018; Zhang et al., 2022). Lactobacillus is well recognized for its immunomodulatory properties in the gastrointestinal tract, acting in part through the inhibition of NF-κB signaling to facilitate the healing of gastric ulcerative lesions (Shen et al., 2018; Yang et al., 2021; Li et al., 2026). Mechanistically, this protective effect is thought to be associated with lactic acid secretion, which enhances the permeability of *H. pylori* cell walls and may thus exert direct antimicrobial activity (Westerik et al., 2018). Moreover, microbiota-targeted interventions have shown promise in modulating host inflammation by enhancing superoxide dismutase (SOD), glutathione (GSH) and CAT activities and protecting against mucosal injury (Hitch et al., 2022). Nevertheless, whether gut microbiota plays a causal role in mediating inflammatory signaling pathways, such as JAK/STAT, in GU remains to be clarified.

Traditional Chinese medicine has long been used to manage the management of gastrointestinal disorders and is attracting increasing attention due to its multi-component and multi-target characteristics (Tian et al., 2025). Chaihuang weikuining (CHWKN) is a classical herbal formula comprising multiple medicinal plants, including *Astragalus membranaceus* and *Bupleurum* species, and has been clinically applied for the treatment of gastric disorders in China (Weng et al., 2023; Guo et al., 2024a; Zheng et al., 2025; Zhang et al., 2025). *Astragalus membranaceus*, a representative herbal medicine and its bioactive constituents have been reported to modulate immune cells and improve gut microbiota composition (Guo et al., 2024a; Weng et al., 2023). The homogeneous astragalus polysaccharide and cycloastragenol, which are isolated from *Astragalus membranaceus*, have been shown to reduce ulcer area and improve mucosal integrity (Zhang et al., 2025; Zheng et al., 2025). *Polygonum cuspidatum* has been reported to exert protective effects in various animal models of acute gastric ulcer. Its antioxidant properties effectively increase the levels of SOD, GSH, and prostaglandin E2, thereby promoting ulcer healing (Kim et al., 2020). Previous studies have suggested that individual components of these herbs possess anti-inflammatory and microbiota-modulating properties (Kim et al., 2020; Zhang et al., 2025; Zheng et al., 2025). However, the therapeutic effects of CHWKN on gastric ulcer and, more importantly, its underlying mechanisms, particularly with respect to gut microbiota, remain largely unexplored.

In this study, we aimed to investigate the protective effects of CHWKN against ethanol-induced GU and to elucidate its underlying mechanisms. Specifically, we hypothesized that CHWKN exerts its gastroprotective effects by modulating gut microbiota, thereby suppressing inflammatory signaling pathways such as JAK1/STAT3. To test this hypothesis, we combined antibiotic-mediated microbiota depletion, fecal microbiota transplantation (FMT), and 16S rDNA sequencing to systematically evaluate the role of gut microbiota in CHWKN-mediated protection. Our findings provide new insights into the microbiota-dependent mechanisms of CHWKN and highlight its potential as a therapeutic strategy for GU.

## 2 Material and methods

### 2.1 Preparation and quantitative evaluation of the CHWKN

*Astragalus membranaceus, Bupleurum chinense, Polygonum cuspidatum, Cyperus rotundus, Codonopsis pilosula, Paeonia, Glycyrrhiza uralensis, Cinnamomum cassia, Zingiber officinale, Ziziphus jujuba*, maltose were purchased from the outpatient department of Zhejiang Chinese University (Zhejiang, China). CHWKN was extracted in 100 mL of distilled water at 100 °C for 1 hour and concentrated (4.8 g/L) via rotary evaporation. The chemical components were analyzed by the UHPLC-QExactive system (Thermo Fisher Scientific, USA). The data were processed by Progenesis QI software (Waters Corporation, Milford, MA, USA). Feature peaks were then annotated by matching both MS and MS/MS spectra against the Majorbio Traditional Chinese Medicine-specific metabolite database (Majorbio, Shanghai, China).

### 2.2 Animal

Male SD rats were purchased from the Shanghai Family Planning Research Institute (Shanghai, China). All animal experiments were approved by the Institutional Animal Care and Use Committee of Zhejiang Chinese Medical University (IACUC-20210726-05). The rats were housed in a specific pathogen-free (SPF) barrier facility under controlled conditions: temperature was maintained at 23 ± 1 °C, and relative humidity at 50%−65%, with free access to food and water.

### 2.3 Gastric ulcer models

Following a 1-week of acclimatization period, the rats were randomly divided into five groups: control group (*n* = 8), model group (*n* = 8), low-dose CHWKN group (*n* = 8), high-dose CHWKN group (*n* = 8), and omeprazole group (*n* = 8). As described previous study (Shu et al., 2026), rats except those in the control group were administered 5 mL/kg absolute ethanol by gavage once before the first administration of CHWKN or other treatments, while the control group received an equal volume of normal saline. Meanwhile, based on clinical equivalent dose conversion and according to the body surface area conversion principle, the low- and high-dose CHWKN groups were treated with 7.5 and 15 g/kg of CHWKN, respectively, and the omeprazole group received 20 mg/kg of omeprazole. After 14 days of treatment, all rats were sacrificed, and blood and gastric tissue were collected.

### 2.4 ABX rat models

Rats were randomly assigned to the model group (*n* = 8), the CHWKN group (*n* = 8), ABX group (*n* = 20) and the CHWKN + ABX group (*n* = 8). Rats in the model and CHWKN groups received sterile deionized drinking water. Rats in the ABX and CHWKN + ABX groups had ad libitum access to drinking water containing vancomycin (0.5 g/L), ampicillin (1 g/L), neomycin (0.5 g/L), and metronidazole (1 g/L) for 2 weeks to establish the ABX model (Vicentini et al., 2021; Rakoff-Nahoum et al., 2004). All antibiotics were purchased from MCE (Shanghai, China).

Fecal samples were collected on day 10 of antibiotic treatment. Bacterial load was assessed by quantitative PCR targeting the bacterial 16S rRNA gene to evaluate the reduction in gut bacteria following ABX treatment (Supplementary Figure 1A). After completion of antibiotic treatment, the 20 rats initially assigned to the ABX group were randomly allocated to the ABX group (*n* = 8), ABX + ethanol + FMT-ethanol group (*n* = 6), and ABX + ethanol + FMT-ethanol-CHWKN group (*n* = 6). FMT was initiated 24 h after discontinuation of antibiotics, as described in Section 2.5.

### 2.5 FMT rat models

Fecal donors comprised ethanol-treated rats (*n* = 6) and rats treated with ethanol and CHWKN (*n* = 6). Both donor groups received their respective treatments for 14 days as described above. Fresh fecal samples were collected from donor rats using sterile saline, at a concentration of 10 mg/mL, soaked for 1 min, and homogenized for 10 min. The homogenate was then centrifuged at 6000 rpm for 15 min, and the supernatant was collected for transplantation. The prepared fecal suspensions were administered to recipient rats by oral gavage at 1 mL per 100 g body weight.

FMT was initiated 24 h after discontinuation of antibiotic treatment and administered once every 2 days for 14 days. The ABX + ethanol + FMT-ethanol group (*n* = 6) received fecal suspension from ethanol-treated donors, whereas the ABX + ethanol + FMT-ethanol-CHWKN group (*n* = 6) received fecal suspension from donors treated with ethanol and CHWKN. The efficiency of gut microbiota restoration was assessed by q-PCR after 10 days of antibiotic treatment (Supplementary Figure 1B).

### 2.6 Biochemical analysis

The serum levels of pepsinogen I (PGI) (ELK Biotechnology, Shanghai, China) and pepsinogen II (PGII)(ELK Biotechnology, Shanghai, China), IL-10 (ELK Biotechnology, Shanghai, China), IL-6 (ELK Biotechnology, Shanghai, China), IL-1β (ELK Biotechnology, Shanghai, China), TNFα (ELK Biotechnology, Shanghai, China), MPO(Nanjing Jiancheng Bioengineering Institute, Nanjing, China), and LPS (Beyotime, Shanghai, China), according to the manufacturer's recommended protocol, were measured by multimode reader (Molecular Devices, USA).

### 2.7 Histopathological analysis

The gastric tissue was fixed in a 4% paraformaldehyde solution, embedded in paraffin wax, and then cut into 4-μm-thick sections. Hematoxylin and eosin staining (H&E) and periodic acid-Schiff (PAS) staining were performed on the sections to evaluate gastric injury and damage to the gastric mucosal barrier.

### 2.8 Real-time PCR

Total RNA was extracted from tissues using the FastPure Complex Tissue/Cell Total RNA Isolation Kit (Vazyme, Nanjing, China), followed by cDNA synthesis using HiScript III RT SuperMix for qPCR (+gDNA wiper) (Vazyme, Nanjing, China) both according to the manufacturer's instructions. Real-time quantitative PCR (qPCR) was performed using a QuantStudio 6 Pro (Thermo Fisher Scientific, USA). The primer sequences are listed in the Table 1.

**Table 1:** Primer sequences.

Gene	Primer sequence
***JAK1***	GGAGGAATGTACTGGGCGTC
	GTCCCTGAAAAGCAGACTGGA
***GAPDH***	TCTCTGCTCCTCCCTGTTC
	ACACCGACCTTCACCATCT
***STAT3***	CACAACCTGCGAAGAATCAAG
	TCTGAACAGATCCACGATCCT

### 2.9 16S rRNA microbiome analysis

We collected feces from rats in the control group (*n* = 6), model group (*n* = 6), and CHWKN-H group (*n* = 6) for 16S rRNA gene sequencing. 16S rRNA gene sequencing was performed by LC-Bio (Hangzhou, China), and the microbiota data were analyzed via the Majorbio Cloud Platform (Shanghai, China). For alpha diversity indices, the Kruskal–Wallis rank-sum test was used for multi-group comparisons, followed by False Discovery Rate correction to control for multiple testing. Beta diversity was evaluated based on Bray–Curtis distances, and group separation was assessed using ANOSIM with 999 permutations. Differential taxa were identified using LEfSe with an all-against-all comparison strategy.

### 2.10 Statistical analysis

All data are expressed as mean ± standard deviation (SD). Statistical analyses were performed using GraphPad Prism 8.0 software (GraphPad Software, San Diego, CA, USA). Differences among multiple groups were evaluated using one-way analysis of variance (ANOVA) followed by Tukey's *post hoc* test for multiple comparisons. Normality and homogeneity of variance were assessed prior to analysis. A value of *P* < 0.05 was considered statistically significant.

## 3 Results

### 3.1 Chemical characterization of CHWKN

To elucidate the chemical basis underlying the pharmacological effects of CHWKN, LC-MS/MS analysis was performed, and representative total ion chromatograms are shown in Figure 1A. As illustrated in Figure 1B, carbohydrates and their derivatives (25%) and amino acids and derivatives (15%) were the predominant components, followed by flavonoids (11%), terpenoids (9%), and phenolic acids (4.5%). Notably, these bioactive compounds are widely reported to possess antioxidant, anti-inflammatory, and microbiota-modulating properties. Since carbohydrates and amino acid derivatives can serve as substrates for gut microbiota (Mora-Flores et al., 2023), while flavonoids and phenolic compounds exert immunomodulatory effects (Al-Khayri et al., 2022), the chemical composition of CHWKN suggests a potential dual role in regulating host inflammation and gut microbial homeostasis. Detailed information on the major constituents is summarized in Table 2.

**Figure 1:**
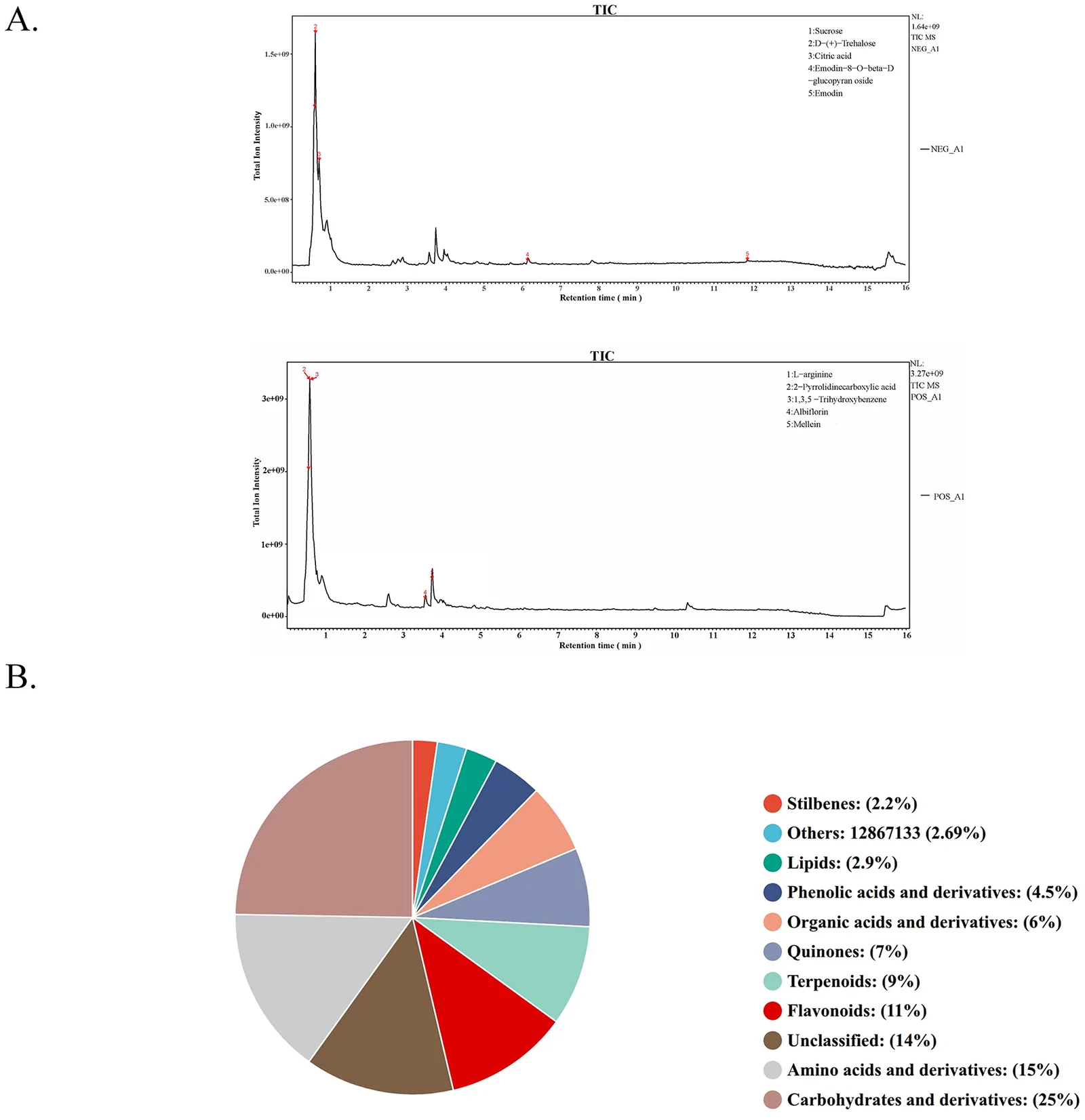
Chemical characterization of CHWKN. **(A)** Representative total ion chromatograms of CHWKN analyzed by LC-MS/MS. **(B)** Classification of identified metabolites, showing the relative abundance of major chemical classes.

**Table 2:** The 10 primary components in CHWKN extract by UHPLC-MS.

NO	Compound name	Average relative content (Mean ±SD)
**1**	D-(+)-Trehalose	14007991.84 ± 388781.36
**2**	L-arginine	7984739.87 ± 168258.01
**3**	Emodin-8-O-beta-D-glucopyranoside	4810380.97 ± 472212.33
**4**	Sucrose	4472581.62 ± 134624.70
**5**	Citric acid	3627162.30 ± 262705.12
**6**	2-Pyrrolidinecarboxylic acid	2732029.91 ± 160254.46
**7**	Albiflorin	2125809.98 ± 203872.30
**8**	Mellein	1990968.62 ± 89850.92
**9**	Emodin	1631300.53 ± 637539.33
**10**	1,3,5-Trihydroxybenzene	1613449.05 ± 217956.71

### 3.2 CHWKN protects against ethanol-induced gastric mucosal injury

To evaluate the gastroprotective effect of CHWKN, as detailed in a previously published study (Huang et al., 2024), an ethanol-induced GU model was established (Figure 2A). Ethanol administration resulted in severe gastric mucosal injury, characterized by hemorrhage, epithelial disruption, and inflammatory cell infiltration. In contrast, CHWKN treatment markedly alleviated these pathological changes, as demonstrated by improved mucosal integrity in H&E and PAS staining (Figure 2B). Quantitative analysis further showed that CHWKN significantly reduced the ulcer index and ameliorated mucosal damage compared with the model group (Figure 2C). Additionally, CHWKN increased serum pepsinogen I level and the PGI/PGII ratio (Figure 2D), indicating improved gastric secretory function and reduced mucosal atrophy. Notably, these protective effects were comparable to those observed in the omeprazole (OME) group, a standard anti-ulcer agent, supporting the therapeutic potential of CHWKN.

**Figure 2:**
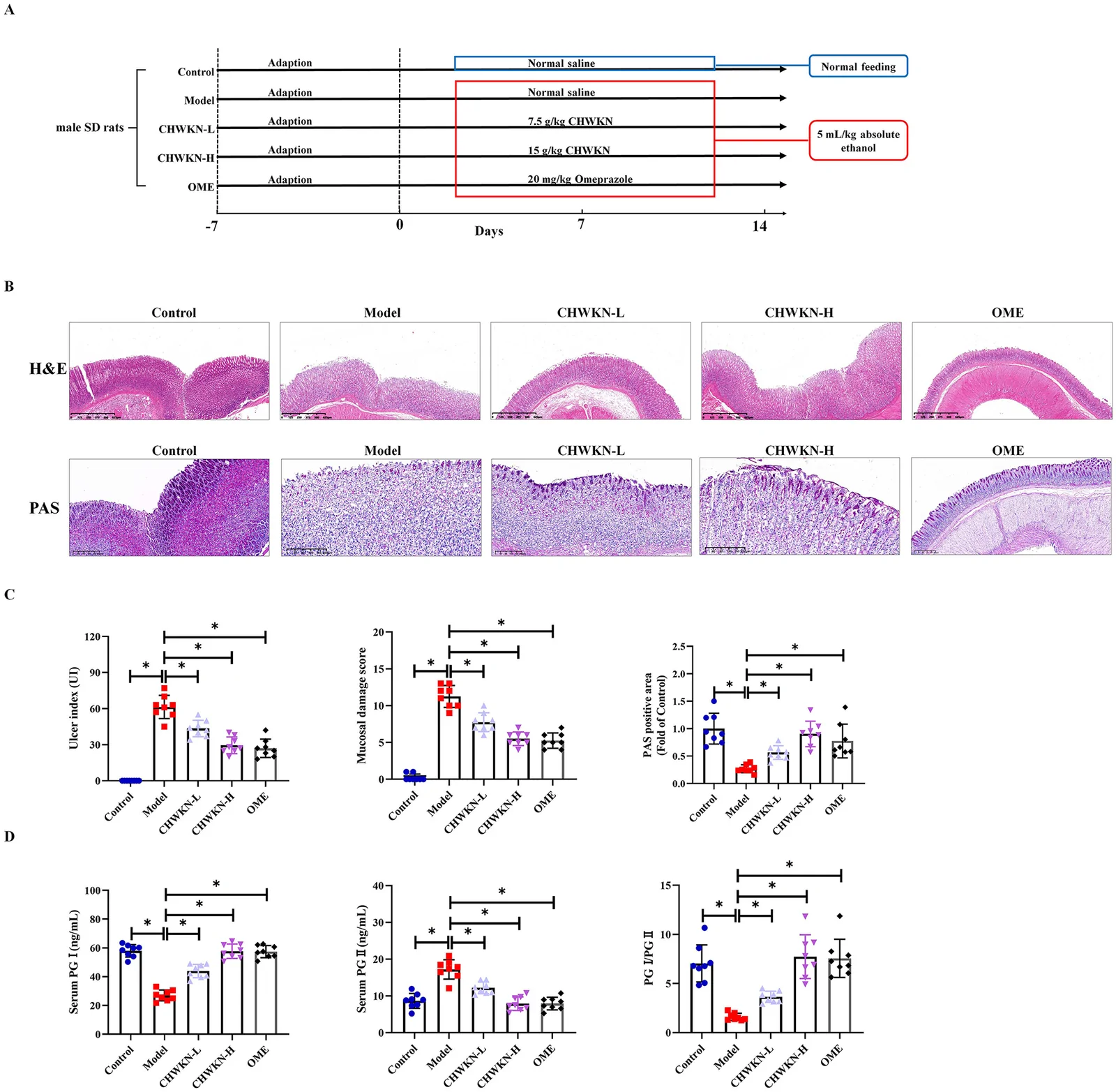
CHWKN protects against ethanol-induced gastric mucosal injury. **(A)** Experimental design of the ethanol-induced gastric ulcer model. **(B)** Representative images of H&E and PAS staining of gastric tissues. **(C)** Quantification of ulcer index and histological damage. **(D)** Serum levels of pepsinogen I (PGI), pepsinogen II (PGII), and PGI/PGII ratio. CHWKN-L, low-dose CHWKN treatment; CHWKN-L, high-dose CHWKN treatment; OME, omeprazole treatment. *P < 0.05 represents statistical significance.

### 3.3 CHWKN suppresses gastric inflammation via inhibition of JAK/STAT3 signaling

To investigate the anti-inflammatory effects of CHWKN, circulating inflammatory mediators were assessed. Exposure to ethanol exposure significantly increased the level of pro-inflammatory cytokines, including IL-1β, IL-6, TNF-α, MPO, and LPS, while decreasing the level of the anti-inflammatory cytokine IL-10 (Figures 3A–F). CHWKN treatment markedly reversed these changes, indicating a robust anti-inflammatory effect. Notably, similar trends were observed in the OME group, which served as a positive control. Given the central role of the JAK/STAT pathway in inflammatory responses (Agashe et al., 2022), we further examined its activation. qRT-PCR analysis showed that ethanol markedly upregulated *Jak1* and *Stat3* expression, whereas both CHWKN and OME treatment significantly suppressed their expression (Figures 3G, H). These findings suggest that CHWKN alleviates ethanol-induced gastric inflammation, at least in part, through inhibition of *JAK1/STAT3* signaling. Given the emerging role of gut microbiota in regulating gastric inflammation, we next investigated whether the protective effects of CHWKN depend on microbiota.

**Figure 3:**
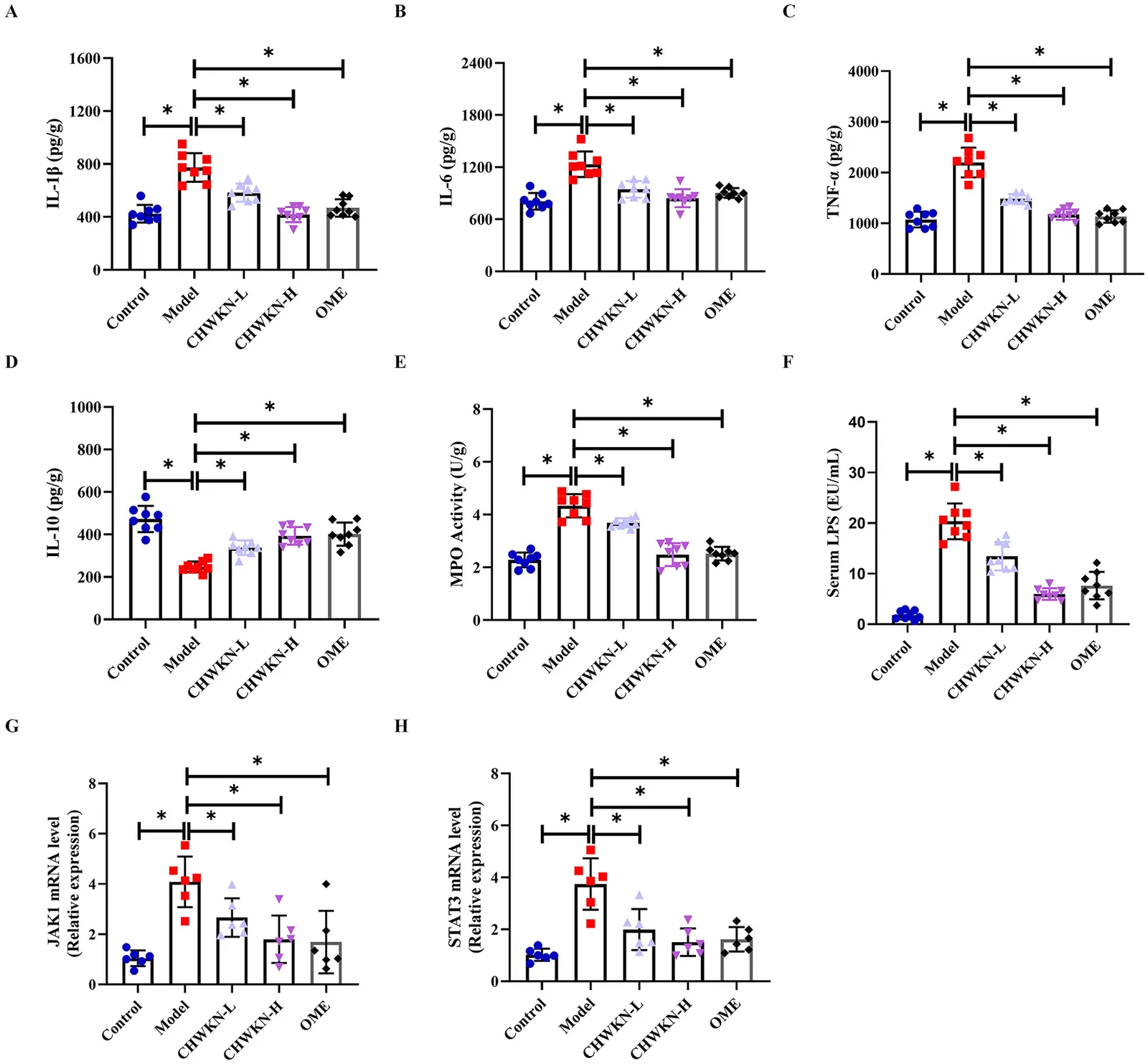
CHWKN suppresses gastric inflammation and *JAK/STAT3* signaling. The levels of serum **(A)** IL-1β; **(B)** IL-6; **(C)** TNFα; **(D)** IL-10; **(E)** MPO; **(F)** LPS; **(G)** The mRNA expression levels of *Jak1*; **(H)** The mRNA expression levels of *Stat3*. CHWKN-L, low-dose CHWKN treatment; CHWKN-L, high-dose CHWKN treatment; OME, omeprazole treatment. *P < 0.05 represents statistical significance.

### 3.4 Gut microbiota is required for the gastroprotective effects of CHWKN

To determine whether gut microbiota contributes to the protective effects of CHWKN, antibiotic-treated (ABX) rats were used to deplete gut microbiota (Figure 4A). Histological analysis revealed that depletion of the microbiota markedly attenuated the protective effects of CHWKN, as evidenced by aggravated mucosal injury, increased epithelial erosion, and enhanced inflammatory infiltration in the ABX+CHWKN group compared with the CHWKN group (Figures 4B, C). Consistently, the beneficial effects of CHWKN on gastric function, as indicated by increased PGI levels and the PGI/PGII ratio, were significantly diminished following microbiota depletion (Figure 4D).

**Figure 4:**
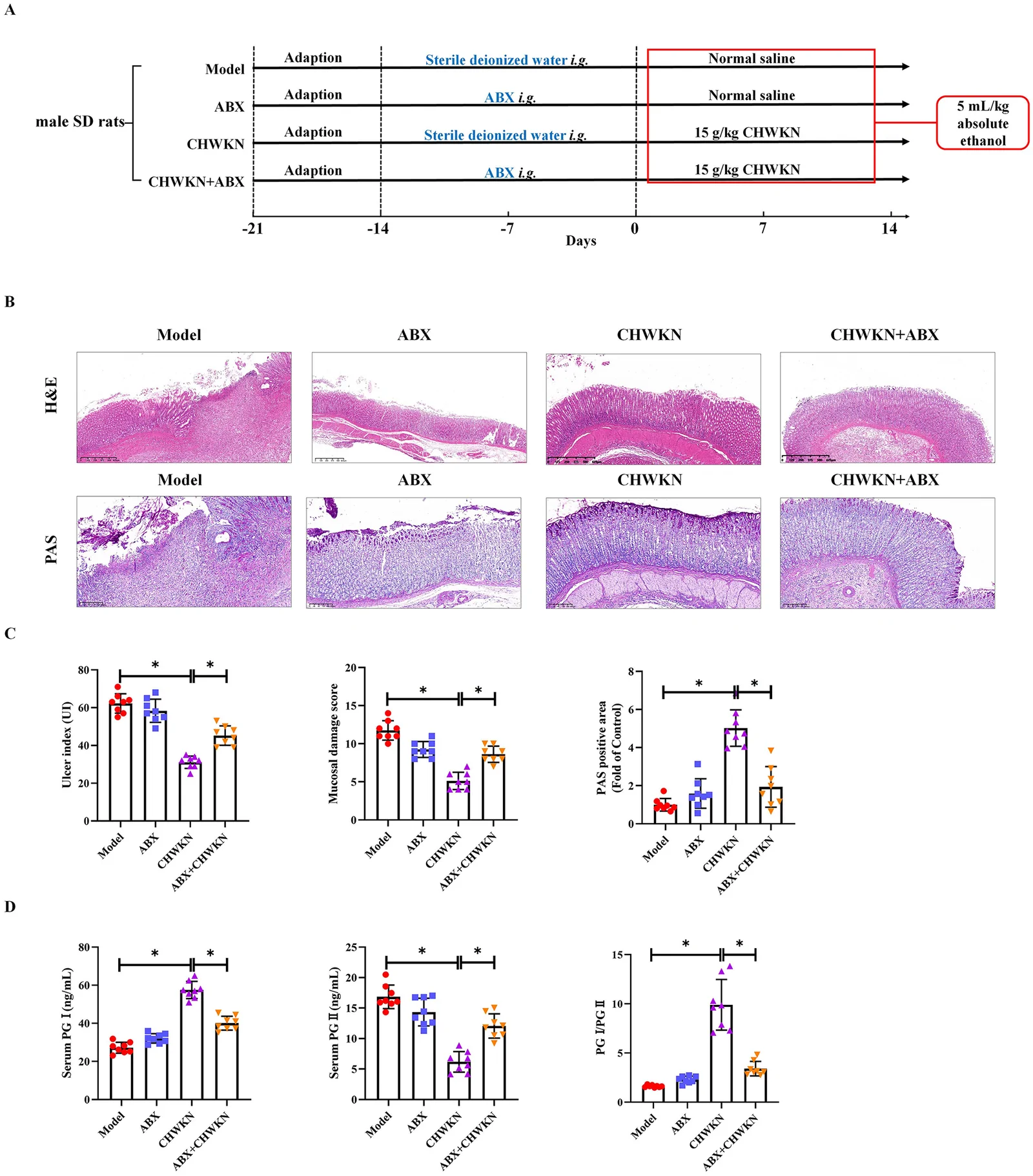
Gut microbiota depletion attenuates the gastroprotective effects of CHWKN. **(A)** Schematic diagram of experimental design; **(B)** Representative image of H&E and PAS staining of rat gastric mucosal; **(C)** Ulcer index, H&E score, and PAS score; **(D)** Serum levels of pepsinogen I (PGI), pepsinogen II (PGII), and PGI/PGII ratio. *P < 0.05 represents statistical significance.

### 3.5 Gut microbiota mediates the anti-inflammatory effects of CHWKN

We next examined whether microbiota depletion affects CHWKN-mediated anti-inflammatory responses. Compared with the CHWKN group, the ABX+CHWKN group exhibited significantly elevated levels of IL-1β, IL-6, TNF-α, MPO, and LPS, accompanied by a reduction in IL-10 levels (Figures 5A–F). Furthermore, the inhibitory effects of CHWKN on *Jak1* and *Stat3* expression were abolished following microbiota depletion (Figures 5G, H). These findings indicate that gut microbiota is essential for the anti-inflammatory effects of CHWKN and its regulation of *JAK1/STAT3* signaling.

**Figure 5:**
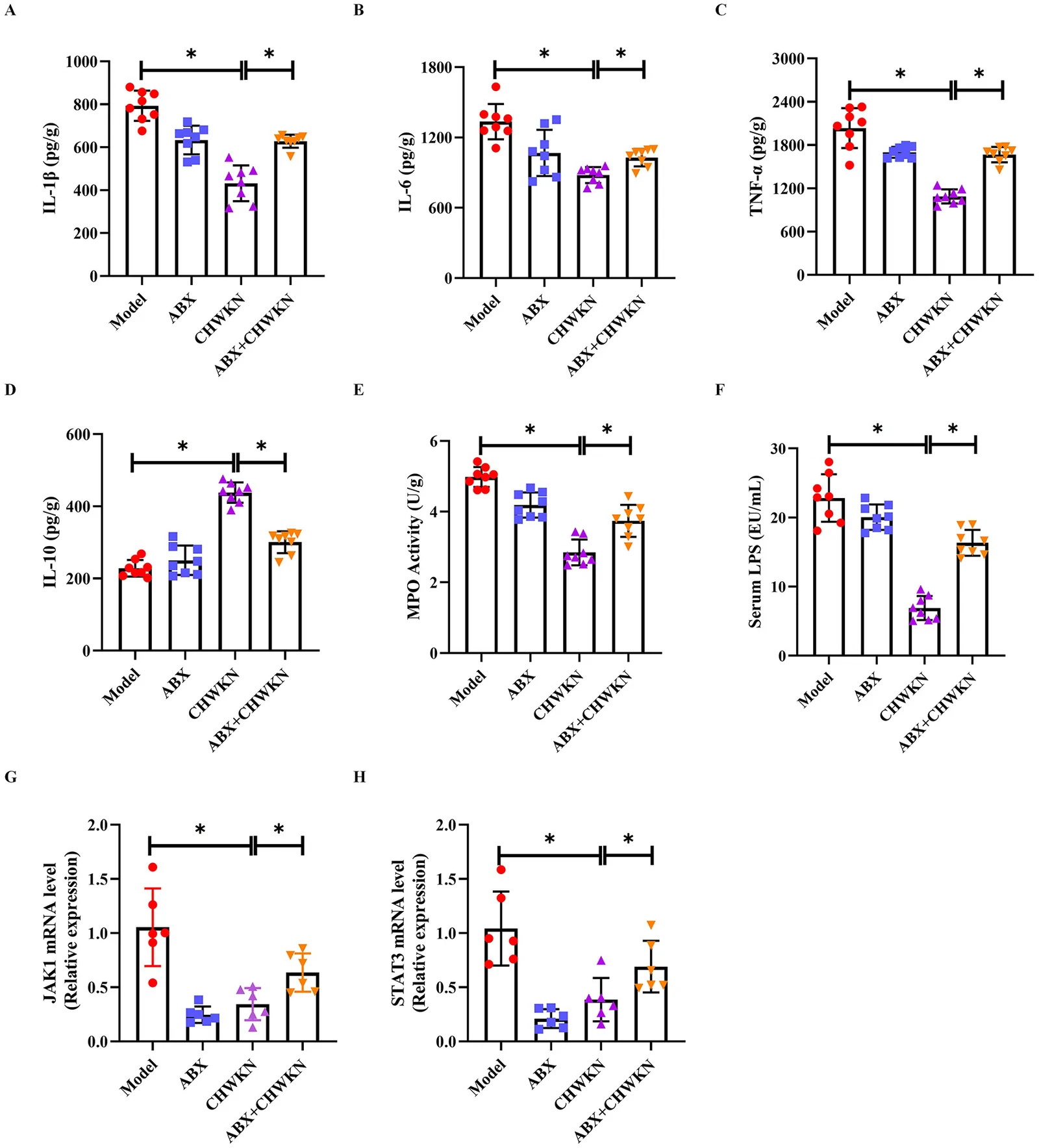
Microbiota depletion impairs the anti-inflammatory effects of CHWKN. The levels of serum **(A)** IL-1β; **(B)** IL-6; **(C)** TNFα; **(D)** IL-10; **(E)** MPO; **(F)** LPS; **(G)** The mRNA expression levels of *Jak1*; **(H)** The mRNA expression levels of *Stat3*. ABX, antibiotic-treated rats; ABX+CHWKN, antibiotic and CHWKN-treated rats. *P < 0.05 represents statistical significance.

### 3.6 CHWKN-associated microbiota is sufficient to confer protection against gastric injury

To further establish the causal role of gut microbiota, FMT was performed (Figure 6A). Rats receiving microbiota from CHWKN-treated donors (the donor rats treated with ethanol and CHWKN, CHWKN-FMT) exhibited significantly improved gastric mucosal integrity compared with those receiving microbiota from model donors, as demonstrated by reduced epithelial damage and inflammatory cell infiltration (Figures 6B, C). In addition, CHWKN-FMT restored gastric secretory function, as evidenced by increased serum PGI levels and PGI/PGII ratio (Figure 6D). Consistently, CHWKN-FMT significantly reduced pro-inflammatory cytokines (IL-1β, IL-6, TNF-α, MPO, and LPS) while increasing IL-10 levels (Figures 7A–F). Moreover, *Jak1* and *Stat3* expression were markedly suppressed in the CHWKN-FMT group (Figures 7G, H). These findings demonstrate that CHWKN-associated microbiota is sufficient to confer protection against gastric injury and inflammation, highlighting a causal role of gut microbiota in mediating the therapeutic effects of CHWKN.

**Figure 6:**
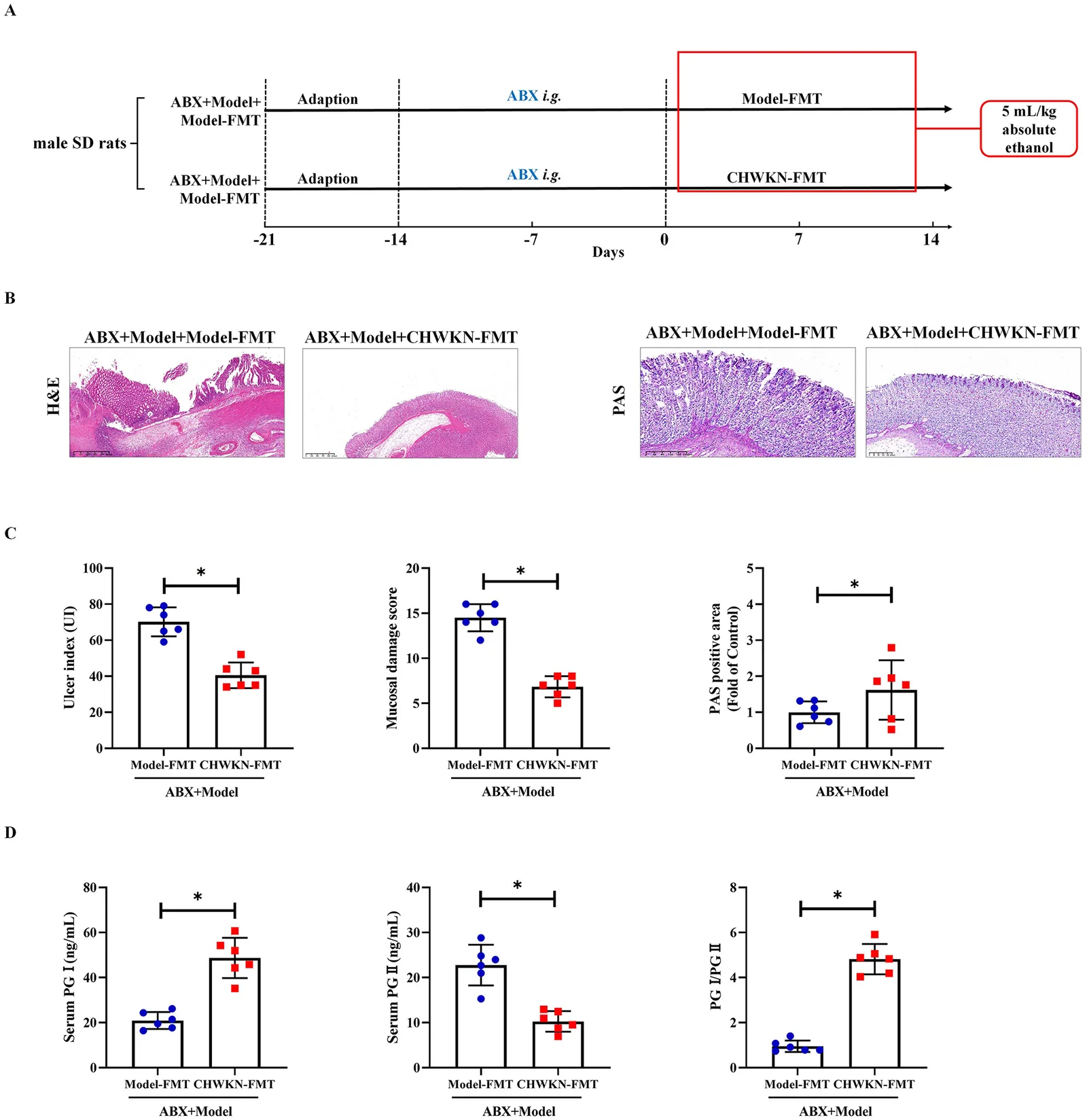
CHWKN-associated microbiota confers protection against gastric injury. **(A)** Experimental design of fecal microbiota transplantation (FMT). **(B)** Representative H&E and PAS staining. **(C)** Quantification of gastric injury. **(D)** Serum PGI, PGII, and PGI/PGII ratio. ABX, antibiotic-treated rats; ABX+CHWKN, antibiotic and CHWKN-treated rats. *P < 0.05 represents statistical significance.

**Figure 7:**
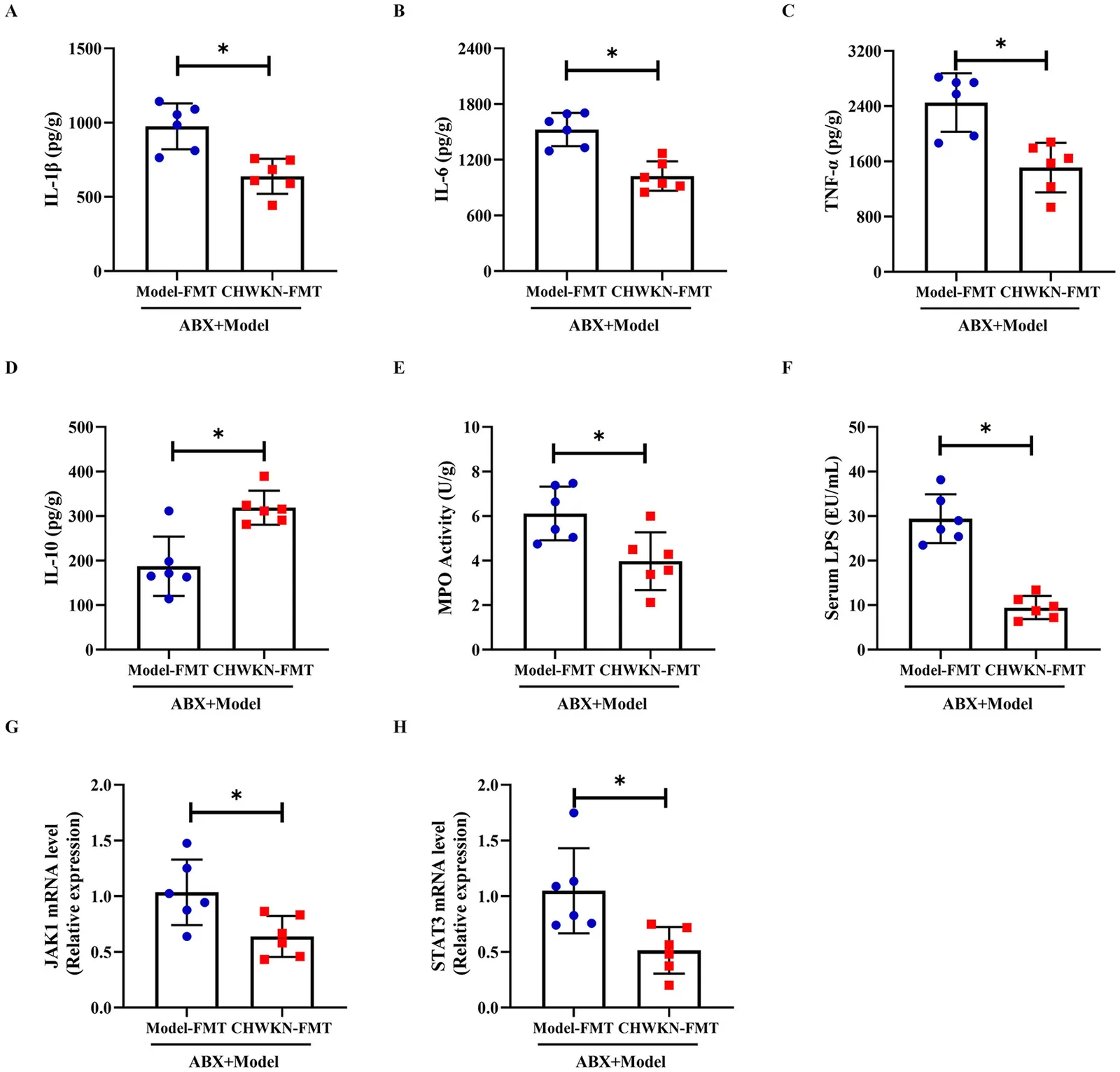
CHWKN-associated microbiota suppresses gastric inflammation. **(A–F)** Serum levels of IL-1β, IL-6, TNF-α, MPO, LPS, and IL-10. **(G and H)** Relative mRNA expression levels of *Jak1* and *Stat3*. *P < 0.05 represents statistical significance.

### 3.7 CHWKN remodels gut microbiota composition in GU

To characterize microbiota alterations, 16S rDNA sequencing was performed. Alpha diversity analysis revealed no significant differences in Chao, ACE, Shannon, and Simpson indices among groups (Figure 8A), suggesting that CHWKN does not significantly impact microbial richness. However, β-diversity analysis (PCoA) showed a clear separation between the model and CHWKN groups on ASV level (Figure 8B), indicating that CHWKN reshapes the structure of the microbial community. LEfSe analysis further exhibited the representative altered by ethanol and CHWKN treatment. The *g_Lachnospiraceae_NK4A136* group, *g_Christensenellaceae_R-7* group, *g_Allobaculum, g_Rothia* were enriched in control group. The genus *g_unclassified*_*f_Anaerovoracaceae, g_unclassified_f_Lachnospiraceae, g_unclassified_f_Oscillospiraceae, g_unclassified_f_Ruminococcaceae, g_norank_f_Desulfovibrionaceae* were enriched in model group. *G_unclassified_f_Prevotellaceae, g_norank_f_Prevotellaceae, g_Dubosiella, g_Faecalibaculum* were enriched in CHWKN group (Figure 8C). Notably, CHWKN treatment increased potentially beneficial bacteria such as *Lactobacillus*, while reducing taxa enriched in the model group. At the genus level, CHWKN partially reversed ethanol-induced dysbiosis, characterized by reduced abundance of *norank_f_Muribaculaceae* and increased *Lactobacillus* (Figure 8D). These results suggest that CHWKN alleviates GU injury, at least in part, by remodeling gut microbiota composition.

**Figure 8:**
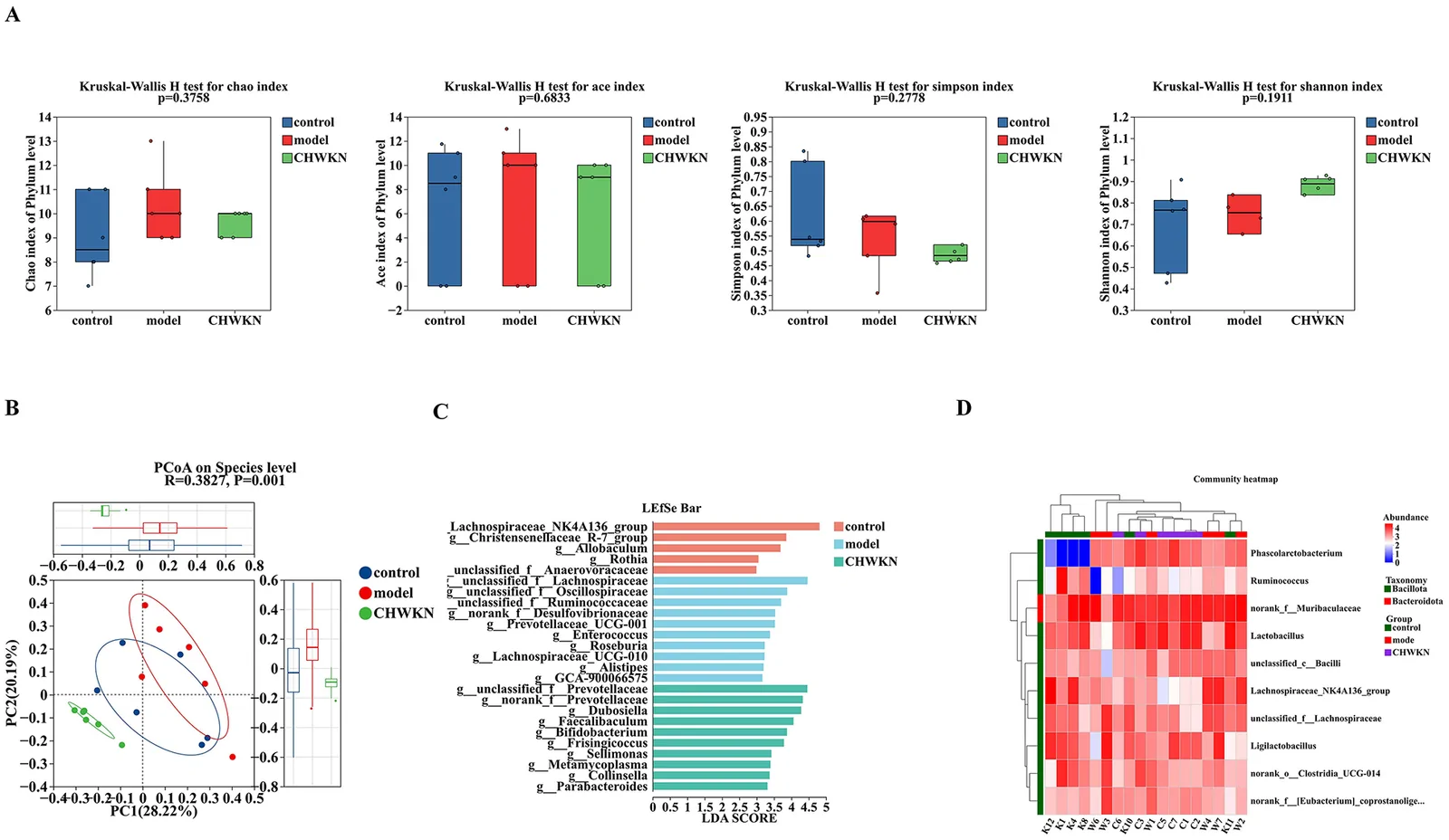
CHWKN remodels gut microbiota composition in gastric ulcer (GU). **(A)** Alpha diversity indices (Chao, ACE, Shannon, Simpson). **(B)** Principal coordinate analysis (PCoA) of microbial communities. **(C)** LEfSe analysis of differential taxa. **(D)** Relative abundance of representative bacterial genera.

## 4 Discussion

In the present study, we demonstrate that CHWKN exerts significant protective effects against ethanol-induced GU. Importantly, our findings reveal that these effects are mediated via a gut microbiota-associated mechanism involving the suppression of inflammatory responses and inhibition of *JAK1/STAT3* signaling. By integrating antibiotic-mediated microbiota depletion, FMT, and microbiome profiling, provides evidence supporting a causal role for gut microbiota in CHWKN-mediated gastroprotection (Figure 9).

**Figure 9:**
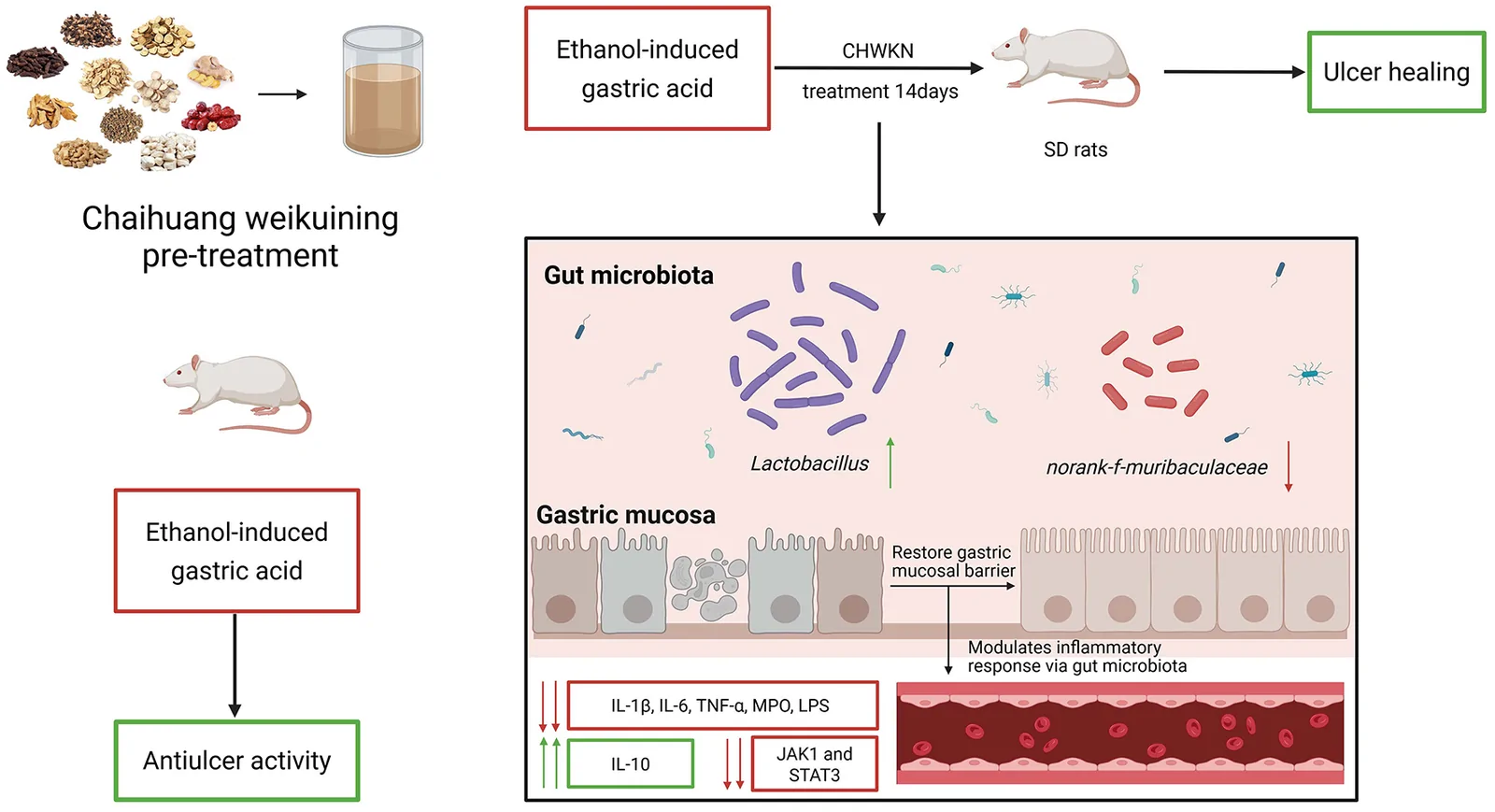
The schematic illustrates the CHWKN against ethanol-induced gastric mucosal injury. Created in BioRender.

Ethanol-induced gastric injury is characterized by disruption of the mucosal barrier disruption, oxidative stress, and excessive inflammatory responses (Zou et al., 2021; Guo et al., 2024b). Consistent with previous reports, we observed that ethanol exposure resulted in severe mucosal damage, accompanied by increased levels of pro-inflammatory cytokines, including IL-1β, IL-6, TNF-α, MPO, and LPS (Guo et al., 2024b; Zhao et al., 2024; Li et al., 2018). These mediators are known to amplify inflammatory cascades and contribute to epithelial injury. Treatment with significantly alleviated these pathological changes, indicating its potent gastroprotective and anti-inflammatory properties. Notably, the effects of CHWKN were comparable to those of omeprazole (Oosterhuis and Jonkman, 1989), a clinically established anti-ulcer agent, further supporting its therapeutic potential. Inflammation plays a central role in GU progression, and the JAK/STAT signaling pathway is a key regulator of inflammatory responses (Agashe et al., 2022). Activation of JAK1/STAT3 has been reported to promote cytokine production and sustain inflammatory signaling in gastrointestinal disorders (Sarapultsev et al., 2023). In this study, CHWKN markedly suppressed the expression of *Jak1* and *Stat3*, suggesting that inhibition of JAK/STAT3 signaling contributes to its anti-inflammatory effects. These findings are consistent with the notion that targeting inflammatory signaling pathways represents an effective strategy for mitigating gastric mucosal injury.

A major finding of this study is the identification of gut microbiota as a critical mediator of the action of CHWKN. Increasing evidence indicates that gut microbiota plays an essential role in maintaining gastrointestinal homeostasis and regulating host immune responses (Yoo et al., 2020; Wang et al., 2024). Dysbiosis has been associated with enhanced inflammatory signaling and impaired mucosal repair (Santana et al., 2022). In the present study, antibiotic-mediated depletion of gut microbiota abolished both the protective and anti-inflammatory effects of CHWKN, demonstrating that microbiota is required for its therapeutic action. More importantly, fecal microbiota transplantation experiments further established the causal role of microbiota. Transfer of microbiota from CHWKN-treated donors was sufficient to confer protection against gastric injury and suppress inflammatory responses in recipient rats. These results provide strong evidence that CHWKN does not merely act on host tissues directly, but rather exerts its effects, at least in part, through modulation of gut microbiota. Such microbiota-associated mechanisms have been increasingly recognized in the pharmacological actions of herbal medicines.

Consistent with these functional findings, 16S rDNA sequencing revealed that CHWKN reshaped gut microbial composition. Although microbial richness was not significantly altered, CHWKN induced clear changes in microbial community structure and increased the abundance of beneficial taxa, such as *Lactobacillus*. We acknowledged only reliance on 16S rRNA sequencing, does not make sure definitively pinpoint the functional bacterial taxa and metabolites mediating the protective effects against ethanol induced gastric injury. Nevertheless, previous study reported *Lactobacillus rhamnosus* GG facilitates gastric ulcer healing by reducing the apoptosis to proliferation ratio and promoting angiogenesis (Lam et al., 2007). In addition, *L. rhamnosus SHA113* and its conditioned medium have been reported to exert anti-inflammatory activity and attenuate ethanol induced mucosal damage (Yang et al., 2021). These data collectively suggest that *Lactobacillus* species may participate in the protective effects to gastric ulcer. However, definitive attribution of causality requires further experimentation, including targeted bacterial depletion or reconstitution studies. Lipopolysaccharide (LPS), an integral component of the outer membrane of Gram-negative bacteria, is a well-recognized pro-inflammatory endotoxin and serves as a reliable indicator of low-grade systemic inflammation (Di Vincenzo et al., 2024). We found serum LPS levels were significantly reduced following CHWKN administration. Notably, this effect was largely abolished by gut microbiota depletion and restored by fecal microbiota transplantation, suggesting that the impact of CHWKN on systemic inflammatory status is mediated, through the gut microbiota. The JAK1/STAT3 signaling pathway is a classical pathway through which LPS exerts its pro-inflammatory effects (Ji et al., 2025). Our findings also suggest a potential link between gut microbiota and JAK1/STAT3 signaling. Therefore, microbiota remodeling induced by CHWKN may contribute to suppression of inflammatory signaling and protection of the gastric mucosa. Our findings also suggest a potential link between gut microbiota and *JAK1/STAT3* signaling. Although the precise mechanisms remain to be fully elucidated, it is plausible that microbiota-derived metabolites or microbial-host interactions may regulate inflammatory pathways, thereby influencing JAK1/STAT3 activation. This microbiota-inflammation signaling axis may represent a key mechanism underlying the therapeutic effects of CHWKN.

Several limitations of this study should be acknowledged. First, although we demonstrated a causal role of microbiota using ABX and FMT approaches, the specific microbial taxa and metabolites responsible for the observed effects remain unclear. Second, the molecular mechanisms linking microbiota alterations to JAK1/STAT3 signaling require further investigation. Future studies incorporating metabolomics and mechanistic analyses will help to clarify these pathways.

## 5 Conclusion

This study demonstrates that in contrast to single compound therapies, CHWKN exerts concurrent modulation of gut microbiota and suppression of the JAK1/STAT3 inflammatory pathway, offering a dual mechanism that addresses both microbial dysbiosis and mucosal inflammation. These findings not only provide mechanistic insight into the therapeutic effects of CHWKN but also highlight the potential of targeting gut microbiota as a strategy for GU management, although further evidence from pharmacokinetic, toxicological, and clinical studies is required to validate this potential. Collectively, these findings position CHWKN as a promising multi-targeted therapeutic candidate for gastric ulcer disease, with potential to serve as an adjunct or alternative to antibiotics and proton pump inhibitors.

## Data Availability

The data analyzed in this study are available in the NCBI SRA (https://www.ncbi.nlm.nih.gov/sra) under BioProject accession PRJNA1483298.

## References

[B1] Agashe R. P. Lippman S. M. Kurzrock R. (2022). JAK: not Just Another Kinase. Mol. Cancer Ther. 21, 1757–1764. doi: 10.1158/1535-7163.MCT-22-0323 36252553 PMC10441554

[B2] Aldriwesh M. G. Alotibi R. S. Alqurainy N. Alrabiah S. Arafah A. M. Alghoribi M. F. . (2026). The role of gut microbiome in aging-associated diseases: where do we stand now and how technology will transform the future. Gut Microbes 18:2607076. doi: 10.1080/19490976.2025.2607076 41454672 PMC12758313

[B3] Al-Khayri J. M. Sahana G. R. Nagella P. Joseph B. V. Alessa F. M. Al-Mssallem M. Q. (2022). Flavonoids as potential anti-inflammatory molecules: a review. Molecules 27:2901. doi: 10.3390/molecules27092901 35566252 PMC9100260

[B4] Arshad S. A. Murphy P. Gould J. C. (2025). Management of perforated peptic ulcer: a review. JAMA Surg. 160, 450–454. doi: 10.1001/jamasurg.2024.6724 39937493

[B5] Chen X. Xia C. Li Q. Jin L. Zheng L. Wu Z. (2018). Comparisons between bacterial communities in mucosa in patients with gastric antrum ulcer and a duodenal ulcer. Front. Cell Infect. Microbiol. 8:126. doi: 10.3389/fcimb.2018.00126 29868487 PMC5952031

[B6] Di Vincenzo F. Del Gaudio A. Petito V. Lopetuso L. R. Scaldaferri F. (2024). Gut microbiota, intestinal permeability, and systemic inflammation: a narrative review. Intern. Emerg. Med. 19, 275–293. doi: 10.1007/s11739-023-03374-w 37505311 PMC10954893

[B7] Guo R. Zhang H. Jiang C. Niu C. Chen B. Yuan Z. . (2024a). The impact of *Codonopsis Pilosulae* and *Astragalus Membranaceus* extract on growth performance, immunity function, antioxidant capacity and intestinal development of weaned piglets. Front. Vet. Sci. 11:1470158. doi: 10.3389/fvets.2024.1470158 39376910 PMC11456569

[B8] Guo Y. Wu Y. Huang T. Huang D. Zeng Q. Wang Z. . (2024b). Licorice flavonoid ameliorates ethanol-induced gastric ulcer in rats by suppressing apoptosis via PI3K/AKT signaling pathway. J Ethnopharmacol 325:117739. doi: 10.1016/j.jep.2024.117739 38301986

[B9] Hitch T. C. A. Hall L. J. Walsh S. K. Leventhal G. E. Slack E. De Wouters T. . (2022). Microbiome-based interventions to modulate gut ecology and the immune system. Mucosal Immunol. 15, 1095–1113. doi: 10.1038/s41385-022-00564-1 36180583 PMC9705255

[B10] Hu X. Li J. Fu M. Zhao X. Wang W. (2021). The JAK/STAT signaling pathway: from bench to clinic. Signal Transduct. Target Ther. 6:402. doi: 10.1038/s41392-021-00791-1 34824210 PMC8617206

[B11] Huang Y. E. Chen S. Y. Li T. J. Tsai Y. S. Chen C. C. . (2024). Gastroprotective effects of Pediococcus acidilactici GKA4 and *Lactobacillus brevis* GKL93 against ethanol-induced gastric ulcers via regulation of the immune response and gut microbiota in mice. Food Funct. 15:11491–11507. doi: 10.1039/D4FO04106B 39480654

[B12] Ji M. Chen M. Zhang N. (2025). Tabersonine inhibits inflammation and apoptosis through the JAK1/STAT3 signaling pathway to alleviate LPS-induced acute lung injury. Sci. Rep. 15:32764. doi: 10.1038/s41598-025-17885-3 40998926 PMC12464169

[B13] Kim Y. S. Nam Y. Song J. Kim H. (2020). Gastroprotective and Healing Effects of *Polygonum cuspidatum* root on experimentally induced gastric ulcers in Rats. Nutrients 12:2241. doi: 10.3390/nu12082241 32727104 PMC7468921

[B14] Lam E. K. Yu L. Wong H. P. Wu W. K. Shin V. Y. Tai E. K. . (2007). Probiotic *Lactobacillus rhamnosus* GG enhances gastric ulcer healing in rats. Eur. J. Pharmacol. 565, 171–179. doi: 10.1016/j.ejphar.2007.02.050 17395175

[B15] Lanas A. Chan F. K. L. (2017). Peptic ulcer disease. Lancet 390, 613–624. doi: 10.1016/S0140-6736(16)32404-7 28242110

[B16] Li Q. Hu X. Xuan Y. Ying J. Fei Y. Rong J. . (2018). Kaempferol protects ethanol-induced gastric ulcers in mice via pro-inflammatory cytokines and NO. Acta Biochim. Biophys. Sin (Shanghai) 50, 246–253. doi: 10.1093/abbs/gmy002 29415150

[B17] Li Y. Jia H. Zhao T. Huo J. Zhang Y. Jiang Y. . (2026). *Lactobacillus rhamnosus* JM023 postbiotic-loaded sodium alginate hydrogel for alleviating alcoholic gastric ulcers. Adv. Healthc. Mater. 15:e71236. doi: 10.1002/adhm.71236 42106931

[B18] Mora-Flores L. P. Moreno-Terrazas Casildo R. Fuentes-Cabrera J. Perez-Vicente H. A. De Anda-Jauregui G. Neri-Torres E. E. (2023). The role of carbohydrate intake on the gut microbiome: a weight of evidence systematic review. Microorganisms 11:1728. doi: 10.3390/microorganisms11071728 PMC10385781 37512899

[B19] Oosterhuis B. Jonkman J. H. (1989). Omeprazole: pharmacology, pharmacokinetics and interactions. Digestion 44, 9–17. doi: 10.1159/000200098 2691315

[B20] Rahman Z. Dwivedi D. K. Jena G. B. (2020). Ethanol-induced gastric ulcer in rats and intervention of tert-butylhydroquinone: Involvement of Nrf2/HO-1 signaling pathway. Hum. Exp. Toxicol. 39, 547–562. doi: 10.1177/0960327119895559 31876185

[B21] Rakoff-Nahoum S. Paglino J. Eslami-Varzaneh F. Edberg S. Medzhitov R. (2004). Recognition of commensal microflora by toll-like receptors is required for intestinal homeostasis. Cell 118, 229–241. doi: 10.1016/j.cell.2004.07.002 15260992

[B22] Santana P. T. Rosas S. L. B. Ribeiro B. E. Marinho Y. De Souza H. S. P. (2022). Dysbiosis in inflammatory bowel disease: pathogenic role and potential therapeutic targets. Int. J. Mol. Sci. 23:3464. doi: 10.3390/ijms23073464 35408838 PMC8998182

[B23] Sarapultsev A. Gusev E. Komelkova M. Utepova I. Luo S. Hu D. (2023). JAK-STAT signaling in inflammation and stress-related diseases: implications for therapeutic interventions. Mol. Biomed. 4:40. doi: 10.1186/s43556-023-00151-1 37938494 PMC10632324

[B24] Shabani M. Ghoshehy A. Mottaghi A. M. Chegini Z. Kerami A. Shariati A. . (2025). The relationship between gut microbiome and human diseases: mechanisms, predisposing factors and potential intervention. Front. Cell Infect. Microbiol. 15:1516010. doi: 10.3389/fcimb.2025.1516010 40395507 PMC12089137

[B25] Shen Z. H. Zhu C. X. Quan Y. S. Yang Z. Y. Wu S. Luo W. W. . (2018). Relationship between intestinal microbiota and ulcerative colitis: mechanisms and clinical application of probiotics and fecal microbiota transplantation. World J. Gastroenterol. 24, 5–14. doi: 10.3748/wjg.v24.i1.5 29358877 PMC5757125

[B26] Shu W. Zhang G. Wu T. Qian J. Zhao Q. Li H. . (2026). A promising traditional Chinese medicine combination derived from Wuji Wan for gastric ulcer therapy by inhibiting ERS through targeting STAT3/PKR to promote autophagy signaling. Phytomedicine 151:157810. doi: 10.1016/j.phymed.2026.157810 41544468

[B27] Tian X. T. Zhan J. P. Qiao C. Ge J. L. Li D. H. (2025). Rising of natural therapies: potential and challenges of traditional Chinese medicine in the management of gastrointestinal diseases. World J. Gastroenterol. 31:103145. doi: 10.3748/wjg.v31.i9.103145 40061595 PMC11886042

[B28] Vicentini F. A. Keenan C. M. Wallace L. E. Woods C. Cavin J. B. Flockton A. R. . (2021). Intestinal microbiota shapes gut physiology and regulates enteric neurons and glia. Microbiome 9:210. doi: 10.1186/s40168-021-01165-z 34702353 PMC8549243

[B29] Wang H. J. Zakhari S. Jung M. K. (2010). Alcohol, inflammation, and gut-liver-brain interactions in tissue damage and disease development. World J. Gastroenterol. 16, 1304–1313. doi: 10.3748/wjg.v16.i11.1304 20238396 PMC2842521

[B30] Wang J. He M. Yang M. Ai X. (2024). Gut microbiota as a key regulator of intestinal mucosal immunity. Life Sci. 345:122612. doi: 10.1016/j.lfs.2024.122612 38588949

[B31] Weng S. Huang L. Cai B. He L. Wen S. Li J. . (2023). Astragaloside IV ameliorates experimental autoimmune myasthenia gravis by regulating CD4 + T cells and altering gut microbiota. Chin. Med. 18:97. doi: 10.1186/s13020-023-00798-6 37542273 PMC10403896

[B32] Westerik N. Reid G. Sybesma W. Kort R. (2018). The probiotic *Lactobacillus rhamnosus* for alleviation of helicobacter pylori-associated gastric pathology in East Africa. Front. Microbiol. 9:1873. doi: 10.3389/fmicb.2018.01873 30154777 PMC6102400

[B33] White J. R. Winter J. A. Robinson K. (2015). Differential inflammatory response to Helicobacter pylori infection: etiology and clinical outcomes. J. Inflamm. Res. 8, 137–147. doi: 10.2147/JIR.S64888 PMC4540215 26316793

[B34] Yan Y. Li B. Gao Q. Wu M. Ma H. Bai J. . (2025). Intestine-decipher engineered capsules protect against sepsis-induced intestinal injury via broad-spectrum anti-inflammation and parthanatos inhibition. Adv. Sci. (Weinh) 12:e2412799. doi: 10.1002/advs.202412799 39836501 PMC11904959

[B35] Yang R. Li J. Xu X. Xu K. Shi J. (2021). Preventive and therapeutic effects of *Lactobacillus rhamnosus* SHA113 and its culture supernatant on alcoholic gastric ulcers. Food Funct. 12, 7250–7259. doi: 10.1039/D1FO00181G 34165119

[B36] Yoo J. Y. Groer M. Dutra S. V. O. Sarkar A. McSkimming D. I. (2020). Gut Microbiota and Immune System Interactions. Microorganisms 8:1587. doi: 10.3390/microorganisms8101587 33076307 PMC7602490

[B37] Zhang D. Xiang M. Jiang Y. Wu F. Chen H. Sun M. (2022). The protective effect of polysaccharide SAFP from *Sarcodon aspratus* on water immersion and restraint stress-induced gastric ulcer and modulatory effects on gut microbiota dysbiosis. Foods 11:1567. doi: 10.3390/foods11111567 35681318 PMC9180856

[B38] Zhang P. P. Tang J. N. Xiang B. Y. Li L. Xie M. Z. Qu H. Y. (2025). Unlocking the potential of Radix Astragali and its active ingredients in gastric ulcer therapy. J. Asian Nat. Prod. Res. 27, 1658–1672. doi: 10.1080/10286020.2025.2475475 40111320

[B39] Zhao M. Qiao C. Yang S. Tang Y. Sun W. Sun S. . (2024). Hinokitiol protects gastric injury from ethanol exposure via its iron sequestration capacity. Eur. J. Pharmacol. 966:176340. doi: 10.1016/j.ejphar.2024.176340 38244759

[B40] Zheng H. Gao S. Liu Y. Wang T. Chen J. Zhang J. . (2025). Bioactive glycyrrhizic acid-astragalus polysaccharide hydrogel facilitates gastric ulcer healing via ROS scavenging and anti-apoptotic effects. Carbohydr. Polym. 362:123685. doi: 10.1016/j.carbpol.2025.123685 40409809

[B41] Zou Y. Cui X. Xiang Q. Guo M. Liang Y. Qu Y. . (2021). Protective effect of against ethanol-induced gastric ulcer and its mechanism. Zhejiang Da Xue Xue Bao Yi Xue Ban 50, 561–567. doi: 10.3724/zdxbyxb-2021-0055 34986535 PMC8732257

