# Supplementary material for: Chaihuang weikuining protects against ethanol-induced gastric ulcer through modulation of gut microbiota and inhibition of JAK1/STAT3 signaling
Source: Front Microbiol. 2026 Oct 27;17:1924725. doi: 10.3389/fmicb.2026.1924725 (PMC13747876; doi:10.3389/fmicb.2026.1924725)
Supplement: Supplementary file 1 [file Data_Sheet_1.docx]

Supplementary Material

## 1. Supplementary Data

## 1.1 Supplementary Table

**Table1. primer sequences**

| Gene | Primer sequence |
| --- | --- |
| *V3-V4* | CCTACGGGNGGCWGCAG |
|  | GACTACHVGGGTATCTAATCC |

## 1.2 Supplementary Figures

**Supplementary Figure 1.** The efficiency of gut microbiota depletion. (A) The efficiency of gut microbiota depletion. (B) The efficiency of gut microbiota restoration.
